# Seroprevalence of human immunodeficiency virus, hepatitis B and C viruses and syphilis infections among blood donors at the Muhimbili National Hospital in Dar Es Salaam, Tanzania

**DOI:** 10.1186/1471-2458-6-21

**Published:** 2006-01-30

**Authors:** Mecky IN Matee, Pius M Magesa, Eligius F Lyamuya

**Affiliations:** 1 Department of Microbiology and Immunology, School of Medicine, Muhimbili University College of Health Sciences, P.O. Box 65001, Dar es Salaam, Tanzania; 2 Department of Hematology and Blood Transfusion, School of Medicine, Muhimbili University College of Health Sciences, P.O. Box 65001, Dar es Salaam, Tanzania

## Abstract

**Background:**

According to the latest Tanzanian National AIDS Control Programme (NACP) report a total of 147,271 individuals donated blood during the year 2002. However, blood safety remains an issue of major concern in transfusion medicine in Tanzania where national blood transfusion services and policies, appropriate infrastructure, trained personnel and financial resources are inadequate. Most of the donated blood is screened for HIV alone.

**Methods:**

We determined among blood donors at Muhimbili National Hospital (MNH), the seroprevalence of human immunodeficiency virus (HIV), hepatitis C virus (HCV), hepatitis B surface antigen (HBsAg) and syphilis by donor type, sex and age and to determine association, if any, in the occurrence of the pathogens. The sample included 1599 consecutive donors, 1424(89.1%) males and 175 (10.9%) females, who donated blood between April 2004 and May, 2005. Most of them 1125 (70.4%) were replacement donors and a few 474 (29.6%) voluntary donors. Their age (in years) ranged from 16 to 69, and most (72.2%) were between 20–39 years.

**Results:**

Two hundred and fifty four (15.9%) of the donated blood had serological evidence of infection with at least one pathogen and 28 (1.8%) had multiple infections. The current seroprevalence of HIV, HBsAg, HCV and syphilis among blood donors at MNH in Dar es Salaam was found to be 3.8%, 8.8%, 1.5% and 4.7%, respectively. Respective seroprevalences among HIV seronegative blood donors were 8.7% for HBV, 1.6% for HCV and 4.6% for syphilis. The differences in the prevalence of HIV and syphilis infections between replacement and voluntary donors were statistically significant (P < 0.05). Syphilis was the only infection that occurred more frequently among HIV infected (12.1%) than non-infected (4.6%) blood donors (P < 0.05), and whose prevalence increased with age (X^2 ^= 58.5 df = 5, P < 0.001). There were no significant sex differences in the occurrence of pathogens. Finally, there were significant associations in the occurrence of HBsAg and syphilis (OR = 2.2, 95% CI 1.1.-4.2) and HIV and syphilis (OR = 2.2, 95% CI 1.0–5.3).

**Conclusion:**

The high (15.9%) seroprevalence of blood-borne infections in blood donated at MNH calls for routine screening of blood donors for HBV, HCV, HIV and syphilis and for strict selection criteria of donors, with emphasis on getting young voluntary donors and for establishment of strict guidelines for blood transfusions.

## Background

The demand for blood transfusion services in Tanzania is high due to endemicity of infections causing anemia, malnutrition, and surgical and obstetrical emergencies associated with blood loss [1,2]. According to the latest National AIDS Control Programme (NACP) report a total of 147,271 individuals donated blood during the year 2002 [1]. However, blood safety remains an issue of major concern in transfusion medicine in Tanzania where national blood transfusion services and policies, appropriate infrastructure, trained personnel and financial resources are inadequate. This is aggravated by the predominance of family and replacement, rather than regular benevolent, non-remunerated donors and lack of comprehensive and systematic screening of donated blood for transfusion-transmissible agents other than HIV. All blood transfusion centres in Tanzania screen donor blood for HIV alone. Other main transfusion transmissible infections such as Hepatitis B and C, malaria, and syphilis are not routinely screened. As a result, some of the blood being transfused is likely to contain unscreened pathogens.

Limited information exists regarding the magnitude of blood-borne pathogens in HIV seronegative donor blood. In a pilot study that we conducted at Muhimbili National Hospital (MNH) in Dar es Salaam in 1999 among 300 blood donors, the overall frequency of anti-HIV, anti-HCV, anti-HBs, HBsAg, anti-HTLV-1, and syphilis antibodies were 8.7%, 8%, 20%, 11%, 0%, and 12.7%, respectively [3]. Among the HIV seronegative donors, the frequency of anti-HCV, anti-HBsAg, HBsAg, anti-HTLV-1, and syphilis antibodies were 8.8%, 22%, 11%, 0%, and 10.9%, respectively. HIV-seropositive donors had an increased risk for being positive for syphilis antibodies and HBsAg, but not anti-HCV, anti-HBsAg or anti-HTLV-1.

However, six years have elapsed since the last study of blood-borne pathogens was conducted [3]. During this time the prevalence of HIV as well as that of HCV, HBsAg, and *T pallidum*, which share common modes of transmission with HIV, are likely to have changed. This scenario is likely to change the risk of transmitting blood-borne pathogens since donor blood is not screened comprehensively for all common blood-borne pathogens. Thus, it is prudent to quantify the risk of blood borne infections associated with such transfusions at regular intervals. In the previous study [3], only a relatively small number of donors was involved (n = 300) and for some investigations such as HCV and HBsAg only 100 donor blood samples were screened due to lack of resources. As a result, the data generated was rather limited. For example, it was not possible to estimate the seroprevalence of the different infections by donor type (replacement or voluntary donor), yet this is thought to be very important information since the prevalence of blood borne pathogens may differ significantly with donor category [4,5]. Furthermore, the small sample size might have undermined the associations in the occurrences of the pathogens. For example, the association between HIV and HCV which was found to be marginally non-significant could have been significant with a larger sample. Since the publication showing the prevalence of HCV to be 8% [3] concerns have been raised about the accuracy of the latex agglutination technique employed in that study. It has been argued that the presented estimate could have been an overrepresentation of the situation emanating from cross-reactivity. More accurate HCV tests have now been developed and are widely available such as enzyme-linked immunosorbent assay (ELISA), radioimmunoassay (RIA) and polymerase chain reaction (PCR). Finally, it is interesting to find out the prevalence of HBsAg, HCV and syphilis infections in HIV seronegative blood, which is normally transfused to the needy patients.

Thus, the objective of this study was to determine, among blood donors at MNH, the seroprevalence of HIV, HCV, HBsAg, and syphilis by donor type, sex and age and to determine association, if any, in the occurrence of infection with the respective pathogens.

## Methods

### Study design and setting

This was a cross sectional study which was carried out in the Departments of Hematology and Blood Transfusion and Microbiology and Immunology in MNH and Muhimbili University College of Health Sciences (MUCHS) in Dar es Salaam between April 2004 and May, 2005.

### Study population

A consecutive sample of 1559 apparently healthy adult voluntary (motivated blood donor, who donates at regular intervals) and replacement (usually one time blood donor only when a relative is in need of blood) blood donors agreed to participate after an informed consent. Individuals who were included in the study were healthy men and non-pregnant non lactating women aged between 18 and 69 years, weighing ≥ 50 kg and with hemoglobin levels above 12.5 g/d1 for females and 13.5 g/d1 for males. Exclusion criteria included: current history of medication and those with a history of operation, serious illness, jaundice, blood transfusion, radiotherapy or any form of cancer therapy. These selection procedures are done routinely in the blood transfusion unit of the department of Hematology and Blood Transfusion, MNH.

### Specimens

Blood samples were collected aseptically in 5 ml red top vacutainers (BD, NJ, USA) and left to clot. Sera specimens were separated after centrifugation, aliquoted into 2 ml cryotubes tubes (Nalge Nunc International, IL, USA) and stored at -20°C until the time for assay.

### Assays

#### HIV serology

HIV status was determined by Vironostika HIV Uni-Form II Ag/Ab (BioMerieux, Boxtel, The Netherlands) and reactive samples were retested by Vironostika HIV Uni-Form II Plus O (BioMerieux, Boxtel, The Netherlands). These assays detect both HIV-1/2 infections. Samples reactive on both tests were considered to be positive for IgG anti HIV antibodies.

#### HCV serology

IgG antibodies to HCV were detected using an ELISA technique (Murex anti-HCV version 4.0). This involved inoculation of diluted sample on microwells coated with highly purified antigens which contained sequences from the core, NS3, NS4 and NS5 regions of HCV. The amount of conjugate bound, and hence colour, in the wells, is directly related to the concentration of antibody in the sample. When the test is run and results are interpreted according to manufacturer's instructions high sensitivity (99.93%) and specificity (99.82%) are achieved.

#### Syphilis serology

Syphilis was diagnosed using VDRL (Murex Diagnostics, Kent, UK) and *Treponema pallidum *Hemagglutination (TPHA) (Fujirebio, Tokyo, Japan). Active syphilis was diagnosed if an individual's blood became positive on both tests.

#### Detection of hepatitis B surface antigen (HBsAg)

HBsAg Version 3.0, an immunoassay, was used for the detection of hepatitis B surface antigen (HBsAg)). The test has sensitivity and specificity of approximately 99.7% and 99.3%, respectively when performed according to the instructions of the manufacturer (Murex Biotech Ltd, Dartford, UK).

### Statistical analysis

Data were coded, entered, cleaned, validated and analysed using SPSS version 12.0 [6]. The seroprevalence of HIV, HCV, HBsAg and syphilis was expressed in percentages for the entire study group and by age, sex and donor category and comparisons between the groups were done using Pearson Chi-Square. Logistic regression was used to determine the associations between the occurrence of HIV, HCV, HBsAg and syphilis after controlling for age and sex. The associations are presented as odds ratio (OR) together with 95% confidence intervals (C1) and were considered to be significant if the corresponding 95% C1 did not include one.

### Ethical issues

A written informed consent was obtained prior to enrollment. The following information was given during donor education to ensure that donors have the information needed to make an informed choice; a complete description of the aims of the study, infectious agents that were being screened, potential benefits and risks, blood collection procedures and assurance of confidentiality of any information given as well as test results. Any other requested additional information was provided to donors by study personnel. Donors who were found have any of the screened pathogens were refereed to the Department of Internal Medicine of the Muhimbili National Hospital where additional investigations and appropriate management and follow-up were given. All donor information and test results were confidentially kept.

## Results

The study recruited a total of 1599 donors, 1424(89.1%) males and 175 (10.9%) females. Most of them 1125 (70.4%) were replacement donors and a few 474 (29.6%) voluntary donors. Their ages (in years) ranged from 16 to 69, mostly (72.2%) were between 20–39 years (Table 1). Two hundred and fifty four (15.9%) of the donated blood had serological evidence of infection with at least one pathogen and 28 (1.8%) had multiple infections. The multiple infections were mostly HBsAg and syphilis, and HIV and syphilis (Table 2). One donor had evidence of infection with HBV, syphilis and HIV. The overall prevalence of HIV, HBsAg, HCV and syphilis were 3.8%, 8.8%, 1.5%, and 4.7%, respectively. The prevalence of syphilis increased with age (X^2 ^= 58.5 df = 5, P < 0.001). There were no significant sex differences. The prevalence of HIV, HBsAg, HCV and syphilis among replacement donors were 4.5%, 9.5%, 1.8% and 6.1%, respectively, while in voluntary donors it was 2.0%, 7.2%, 0.8% and 1.5%, respectively. The differences in the prevalence of HIV and syphilis infections between replacement and voluntary donors were significantly (P < 0.05) (Table 1). The HBsAg and HCV were also more prevalent among replacement donors but the difference was not statistically significant. The prevalence of HBsAg, HCV and syphilis among HIV infected donors was 8.6%, 0.0%, and 12.1%, respectively, compared with 8.7%, 1.6% and 4.6% for HIV-seronegative donors. Only syphilis occurred more frequently among HIV seropositive (12.1%) than HIV seronegative (4.6%) donors (P non-infected individuals (P < 0.05) (Table 1). There were statistically significant associations in the occurrence of HBsAg and syphilis OR = 2.2 (95%CI: 1.1–4.2) and HIV and syphilis OR = 2.2 (95%CI: 1.0–5.3).

## Discussion

The aim of this study was to determine the seroprevalence of HIV, HBsAg, HCV and syphilis among blood donors at MNH in Dar es Salaam, by donor type, age, and sex and to determine association if any, in occurrence of the pathogen as well as potential risk of HBsAg, HCV and syphilis infection associated with HIV seronegative blood transfusion. Most of the donor were males 1424 (88.4%), aged between 20–39 years (72.2%) and were mainly replacement 1125 (69.8%) rather than voluntary donors 474 (29.4%), which is consistent with observations in several other studies in Africa [7-9]. This study showed that 15.9%, (~one out six), of the donated blood was seropositive for at least one of the screened pathogens, which is very high prevalence that calls for strict screening of donated blood and stringent donor selection criteria [4]. It was also noted that 28 (1.8%) of donated blood had serological evidence of multiple infections, most frequently HBsAg and syphilis followed by HIV and syphilis. These three pathogens are the commonest in this donor population and should be always screened for as a matter of priority.

The overall seroprevalence of antibodies against HIV, HBsAg, ant HCV and syphilis were 3.8%, 8.8%, 1.5% and 4.7%, respectively. These figures compared well with those reported in other parts of Africa [4,5,15-17].

Results this study when compared with those of a study conducted in 1999 at the same hospital, which found the prevalence of HIV, HBsAg, HCV and syphilis to be 8.7%, 11.0%, 8% and12.7% respectively [3], represent a reduction in all the screened pathogens, especially HCV and syphilis. The reduction in HIV seem to fit a pattern reported by NACP showing a decrease of HIV infections among blood donors from 33% in 1999 to 10% in 2003 [1]. This could be due to increased self-selection of individuals donating blood, which has been associated with a reduction of number of donors per month, both replacement and voluntary donors, from 744 in 1999 to 495 in 2005 (unpublished observation), which may also explain the reduction in the seroprevalence of HBsAg and syphilis. It is possible that behavioural change, in particular in the youth Tanzania population, may have contributed to the observed decline in the prevalence of HIV and other STIs as suggested by others [18,19]. The high prevalence of HCV reported in 1999 was possibly due to the latex technique used for diagnosis, which has since been found to be unreliable due cross- reactivity giving rise to false positive reactivities [3]. The current prevalence of HCV found in the present study is in keeping with findings in other parts of Africa, showing a range of between 0.2% and 3.0% [4,10,11].

The prevalence of HIV and syphilis among replacement donors was significantly higher than that found in voluntary donors, being 4.5% versus 2.0% for HIV, and 6.1% versus 1.5% for syphilis. A similar tendency was also noted for HBsAg and (9.5% versus 7.5%) for HBsAg, and HCV 2.0% versus 0.8%, though the differences were not statistically significant. These results, which are in keeping with those of other studies [8-10], strongly indicate that replacement donors are less suitable and that major emphasis should be made to encourage voluntary donors.

It is extremely important to note the high prevalence of HBsAg (8.7), HCV (1.6%) and syphilis (4.6%) and among HIV seronegative blood, which is normally deemed, fit for transfusion. These figures, which can be utilized to estimate the risk of transfusion associated transmission HBV, HCV and syphilis, should serve as a remainder to health personnel to take the necessary precautions, including reducing the number of unnecessary transfusions [2,12].

With regard to the occurrence of the pathogens, there were significant associations between HIV and syphilis and syphilis and HBsAg (Table 2). These could be due to the fact that these pathogens are sexually transmitted, especially syphilis which has lesions that promote transmission of HIV infection [13]. The lack of association of the pathogens with HCV could lend support to the argument that this virus is not efficiently transmitted by sexual intercourse, and its epidemiology, in the studied donor population is not linked to that of HIV, HBV or syphilis. Previous studies in Tanzania have not been able to depict the modes of HCV transmission in this country [3,14], a gap that warrants further studies.

## Conclusion

This high (15.9%) seroprevalence of transfusion-transmissible infections in blood donated at MNH is alarming and calls for i) comprehensive screening of donor blood for HIV, HBV, HCV and syphilis ii) strict selection of donors, with emphasis on getting young voluntary non-remunerated donors rather than replacement donors, iii) establishment of strict guidelines for blood transfusions and iv) search for viral co-infections in HIV infected patients.

## Competing interests

The author(s) declare that they have no competing interests.

## Authors' contributions

MIM designed the study and together with EFL and PMM supervised laboratory work. Finally, all authors participated, read and approved the final manuscript.

## Pre-publication history

The pre-publication history for this paper can be accessed here:



## Figures and Tables

**Table 1:** Age, sex, donor category and test results of the individuals who donated blood (n = 1599)

Number tested and percentage seropositive					
	Total sample	HIV	HBsAg	HCV	Syphilis

***Age***					
16–19	109 (7.0%)	107 (2.8%)	109 (8.3%)	109(3.7%)	109(0.0%)
20–29	629 (40.3%)	611 (2.3%)	629(9.2%)	628(0.6%)	629(2.1%)
30–39	497 (31.9%)	487(4.7%)	496(9.7%)	497(2.2%)	495(5.7%)
40–49	234 (15.0%)	224(6.3%)	234(7.3%)	234(1.7%)	234(6.8%)
50–59	78(5.0%)	76(6.6%)	78(10.3%)	78(1.3%)	78(19.2%)
60–69	12(0.8%)	12(0.0%)	12(8.3%)	12(0.0%)	12(16.7%)***
***Sex***					
Males	1424 (89.1%)	1422(3.8%)	1423(9.1%)	1424(1.5%)	1422(4.8%)
Females	175 (10.9%)	175(4.0%)	175(6.3%)	174(1.1%)	175(4.0%)
***Donor category***					
Replacement donors	1125 (70.4%)	1116(4.5)*	1124(9.5%)	1125(1.8%)	1123(6.1%)*
Voluntary donors	474 (29.6%)	441(2.0%)	474(7.2%)	473(0.8%)	474(1.5%)
***HIV serostatus***					
HIV seropositive donors			58(8.6%)	0(0.0%)	58(12.1%)**
HIV seronegative donors			1495(8.7%)	1572(1.6%)	1494(4.6%)
***Total***	1599 (100%)	1557(3.8%)	1598(8.8%)	1597(1.5%)	1597(4.7%)

Test results are not available for all donors

*Significantly higher prevalence among replacement donors (P < 0.05)

**Significantly higher prevalence among HIV seropositive donors (P < 0.05)

*** Significant age differences in the prevalence of syphilis (P < 0.0001)

**Table 2:** Association in the occurrence of HBsAg, HIV, HBV and syphilis determined by logistic regression after adjusting for age and sex and expressed as odds ratio (OR) with 95 percentage confidence interval (95% CI)

Infections	N	%	Crude OR(95% CI)	Adjusted OR (95% CI)
HBsAg and HIV	5	17.9	1.0(0.4–2.5)	1.0(0.4–2.5)
HBsAg and syphilis	11	39.3	2.1(1.1–4.0)	2.2(1.1–4.2)
HIV and syphilis	7	25	2.9(1.3–6.6)	2.2(1.0–5.3)
HCV and syphilis	2	7.1	0.9(0.1–6.6)	0.9 (0.1–6.8)
HBsAg and HCV	2	7.1	0.9(0.2–4.3)	0.9 (0.2–3.9)
